# Divergent Sapovirus Strains and Infection Prevalence in Wild Carnivores in the Serengeti Ecosystem: A Long-Term Study

**DOI:** 10.1371/journal.pone.0163548

**Published:** 2016-09-23

**Authors:** Ximena A. Olarte-Castillo, Heribert Hofer, Katja V. Goller, Vito Martella, Patricia D. Moehlman, Marion L. East

**Affiliations:** 1 Leibniz Institute for Zoo and Wildlife Research, Alfred-Kowalke-Strasse 17, D-10315, Berlin, Germany; 2 Department of Veterinary Medicine, University of Aldo Moro of Bari, S.p. per Casamassima km 3, 70010 Valenzano, Bari, Italy; 3 EcoHealth Alliance, 460 West 34th St, New York, NY, United States of America; Universidade Federal de Minas Gerais, BRAZIL

## Abstract

The genus *Sapovirus*, in the family *Caliciviridae*, includes enteric viruses of humans and domestic animals. Information on sapovirus infection of wildlife is limited and is currently lacking for any free-ranging wildlife species in Africa. By screening a large number of predominantly fecal samples (n = 631) obtained from five carnivore species in the Serengeti ecosystem, East Africa, sapovirus RNA was detected in the spotted hyena (*Crocuta crocuta*, family Hyaenidae), African lion (*Panthera leo*, family Felidae), and bat-eared fox *(Otocyon megalotis*, family Canidae), but not in golden or silver-backed jackals (*Canis aureus* and *C*. *mesomelas*, respectively, family Canidae). A phylogenetic analysis based on partial RNA-dependent RNA polymerase (RdRp) gene sequences placed the sapovirus strains from African carnivores in a monophyletic group. Within this monophyletic group, sapovirus strains from spotted hyenas formed one independent sub-group, and those from bat-eared fox and African lion a second sub-group. The percentage nucleotide similarity between sapoviruses from African carnivores and those from other species was low (< 70.4%). Long-term monitoring of sapovirus in a population of individually known spotted hyenas from 2001 to 2012 revealed: i) a relatively high overall infection prevalence (34.8%); ii) the circulation of several genetically diverse variants; iii) large fluctuations in infection prevalence across years, indicative of outbreaks; iv) no significant difference in the likelihood of infection between animals in different age categories. The likelihood of sapovirus infection decreased with increasing hyena group size, suggesting an encounter reduction effect, but was independent of socially mediated ano-genital contact, or the extent of the area over which an individual roamed.

## Introduction

Knowledge of wildlife pathogens is relatively limited because research is mostly focused on pathogens that threaten the health of humans, livestock and companion animals [1, 2, 3]. Interactions between pathogens and their wildlife hosts are often complex [4, 5, 6, 7] and require long-term interdisciplinary research to unravel [8, 9, 10]. Human activities can disrupt host- pathogen dynamics [11] and reduce the resilience of wildlife to infection [12, 13, 14]. Hence long-term monitoring of wildlife pathogens from ecosystems where the impact of humans is still relatively small provides a useful benchmark to gage future human-induced changes [15].

*Sapovirus* is a genetically diverse genus in the family *Caliciviridae*. Sapoviruses are single-stranded, positive-sense, non-enveloped RNA viruses [16], first detected in humans in the United Kingdom in 1976 [17]. Sapovirus infection in humans is associated with either sporadic cases of gastro-enteritis in children or community-wide outbreaks [16–18]. In the domestic pig (*Sus scrofa domesticus*), the virus is associated with weaning and post-weaning enteritis [19]. The genetic diversity and recombination events observed in sapoviruses may account for their emergence [20].

Sapoviruses are classified into five recognized genogroups (GI-GV), based on the capsid gene (VP1) sequence [21]. Genogroups GI, GII, GIV and GV predominantly comprise human sapoviruses [16]. Most porcine sapoviruses belong to genogroup GIII [21]. Sapoviruses closely related to human variants in genogroup GV have been reported from domestic pigs and the California sea lion (*Zalophus californianus*) [22, 23], suggesting a considerable potential for cross-species transmission [19]. Divergent sapoviruses not yet classified into recognized genogroups are known from pigs [24], farmed American mink (*Neovison vison*) [25], domestic dogs (*Canis lupus familiaris*) [26], brown rats *(Rattus novergicus)* [27] and the Pomona leaf-nosed bat (*Hipposideros pomona*) [28].

Information on sapovirus infection prevalence or genetic diversity in free-ranging wildlife is scant and long-term studies on any wildlife host are lacking. The only free-ranging wild carnivore reported with sapovirus infection that we are aware of is the Californian sea lion [23]. In Africa, sapovirus infection has been reported in humans in several countries [29–31] and human sapoviruses have been detected in sewage-polluted wastewater [32].

The Serengeti National Park (NP), in Tanzania, spans most of the Serengeti ecosystem [33]. Its wild carnivore guild of 26 species [33] provides a multi-host landscape for viral infections [34–36] in which the spotted hyena (*Crocuta crocuta*, family Hyaenidae) is the most abundant large carnivore host [37, 38]. Long-term monitoring of spotted hyenas has revealed that this species is host to several viruses [35, 36, 39, 40] and that pathogen infection can be significantly influenced by social and demographic factors, and life history traits [35, 40–44].

**Fig 1:**
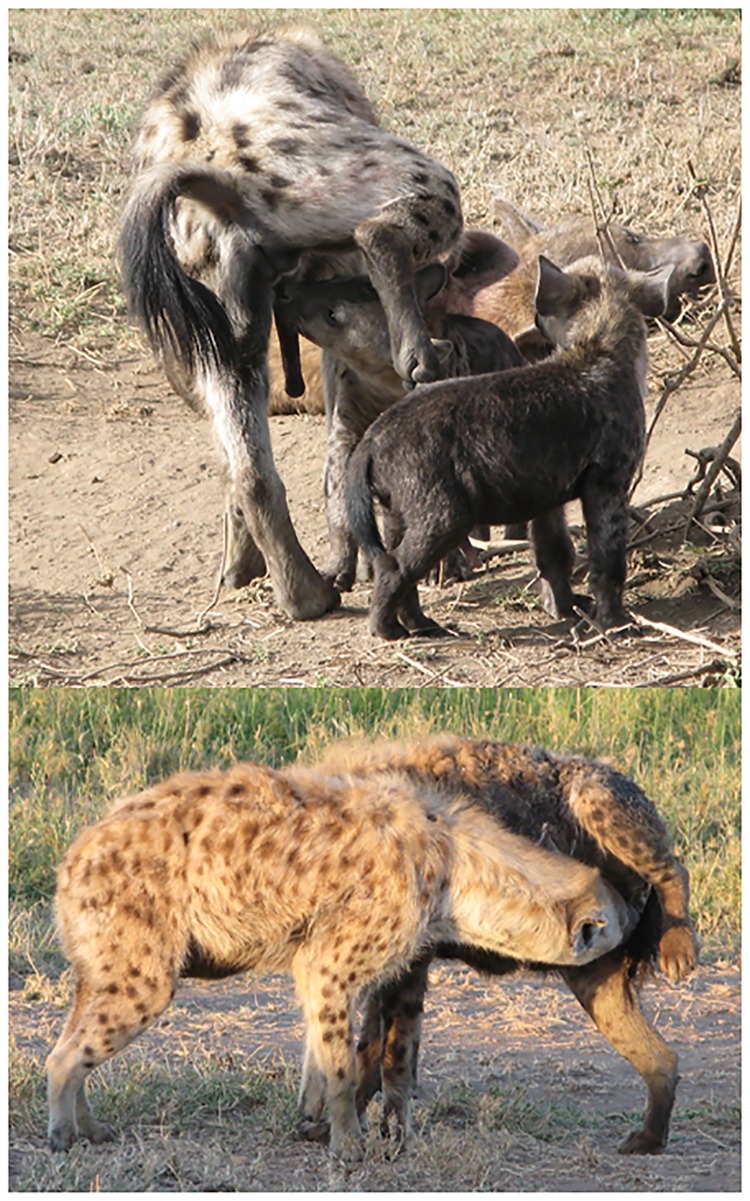
Ritualized greeting ceremonies between members of a spotted hyena clan.

The spotted hyena is a carnivore that forms large, fission-fusion groups, termed clans [38]. Given that enteric viruses, such as sapoviruses, are typically transmitted by the fecal-oral route [16,18] we investigated the effect of three factors (social contact, range size and clan size) that should modulate fecal-oral transmission and hence sapovirus infection risk in spotted hyenas. Spotted hyenas have a ritualized greeting ceremony (Fig 1) during which participants typically sniff and lick each other’s ano-genital region thereby facilitating fecal-oral virus transmission from infected to susceptible clan members. High ranking adult females and their offspring are more often involved in greeting ceremonies than are low ranking adult females and their offspring, and all adult females and their offspring are more often involved in greeting ceremonies than immigrant males [45]. This predicts that ‘social contact’ mediated transmission should be highest in high ranking females and their offspring, intermediate in low ranking females and their offspring, and lowest among immigrant males, all else being equal.

Environmental contamination might be an important route for fecal-oral transmission of sapovirus, for example when spotted hyenas sniff virus infected feces or ingest water contaminated with virus infected feces. If so, individuals with a limited range may be less likely to encounter virus infected feces than those with an extensive range. In the Serengeti NP, adult and subadult hyenas (i.e. those ≥12 months of age) not only range throughout the approximately 56 km^2^ of their clan’s territory, but also undertake long distance foraging trips (of approximately 140 km distance round-trip) from their clan territory [46, 47]. The ranges of cubs (<12 months of age) are by comparison extremely limited, being restricted to the communal den area within the clan territory [47]. If the extent of an animal’s range determines its chance of encountering virus infected feces, then all else being equal, cubs should be less often infected with sapovirus than older animals.

Finally, sapovirus transmission might depend on basic population parameters such as population density, principally represented by clan (group) size. If the chance of transmission increases with animal density, then individuals living in larger clans should be more likely to be infected than those in smaller clans. However, if an encounter reduction effect operates [48, 49], then we expect the chance of susceptible individuals encountering an infected animal to decline with clan size.

In humans, sapovirus infection is currently thought to provide immunological protection, at least to antigenically homologous sapoviruses, although specific immunological responses are still unknown [16]. Currently nothing is known about the immunological responses of spotted hyenas to sapovirus infection, or the length of immunological protection following sapovirus infection. Even so, if sapovirus infection induces long-term immunity against re-infection regardless of strain-type, we would expect cubs (i.e. naïve animals) to be more prone to infection than adults, as is the case for coronavirus infection in this species [35]. However, if sapovirus infection provides only short-term immunity, we would expect re-infections among animals of all ages. If immune responses are strain-specific, re-infection would also be expected in animals of all ages, following the appearance of a divergent strain.

This study aims to advance knowledge of sapovirus infection in wild carnivore communities in Africa. We report the identification of sapoviruses in wild carnivores in Africa and investigate the genetic diversity of strains infecting sympatric carnivore species in the Serengeti ecosystem. We assess temporal changes in sapovirus infection in a large population of individually known spotted hyenas during a period spanning more than a decade and investigate whether sapovirus infection provides long-term immunity against future infection. Furthermore, we test three mechanisms likely to affect the fecal-oral spread of sapovirus infection in spotted hyenas.

## Materials and Methods

### Sample collection

**Fig 2:**
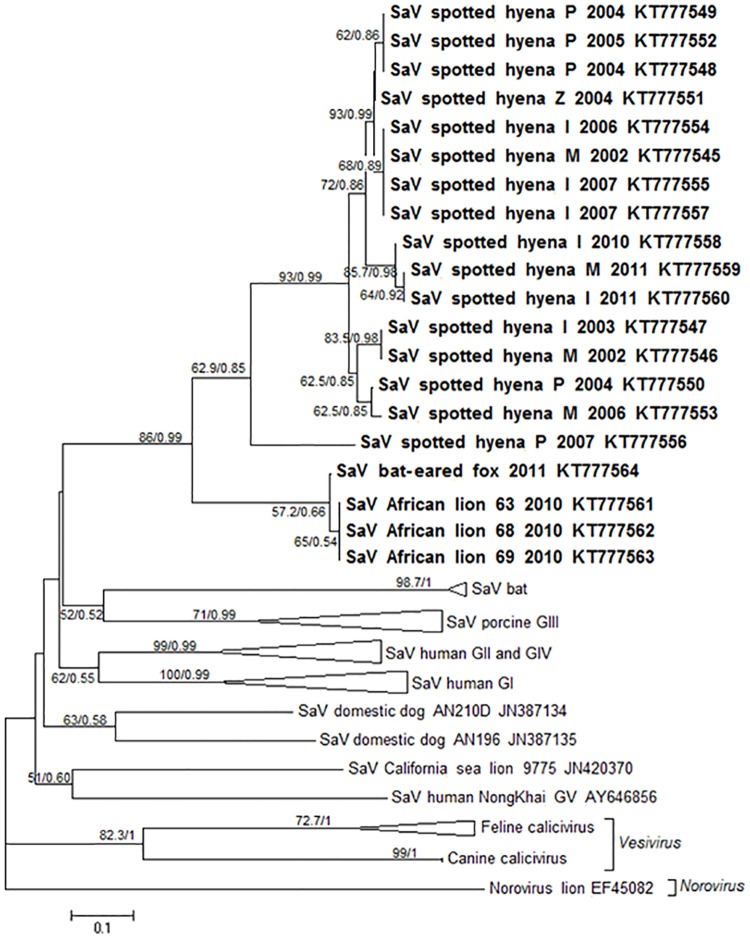
Phylogenetic relationship of sapovirus strains from wild carnivores in the Serengeti National Park. Maximum likelihood (ML) phylogeny under the GTR+I+G model for a fragment of 210 nucleotides of the RdRp gene depicting the relationships between sapovirus (SaV) strains from spotted hyena, bat-eared fox and African lions (in bold) with other strains of the family *Caliciviridae*. The details for strains from the Serengeti National Park include the host species, year of collection and for spotted hyena strains, clan membership of the host (I, M, P) and Z when clan membership was unknown. Numbers at the nodes indicate bootstraps from 1000 replicates/ Bayesian posterior probabilities.

This study was conducted in the Serengeti NP, from February 2001 to March 2012. Fresh fecal samples (n = 514) were collected shortly after deposition from individually known spotted hyenas including 146 samples from adults (females n = 93, males n = 53), 41 samples from subadults (females n = 20, males n = 21) and 327 samples from cubs (females N = 152, males N = 175) from three large clans (denoted in Fig 2 as I, P, and M). Fecal samples were also collected from other carnivores in the Serengeti NP (African lion, *Panthera leo*, N = 9; bat-eared fox, *Otocyon megalotis*, n = 9; silver-backed jackal, *Canis mesomelas*, n = 74 samples; golden jackal, *Canis aureus*, n = 25). Following collection, feces were thoroughly mixed and divided in aliquots. Tissue samples (5 intestine, 2 liver, 9 lung, 10 lymph node, 12 spleen, 10 blood, 1 muscle, 1 saliva) were also collected opportunistically from dead spotted hyenas which were mostly killed by lions or when hit by motor vehicles [40] and hence were not necessarily members of study clans, and from two other carnivore species (bat-eared fox: 2 intestines; silver-backed jackal: 1 intestine, 2 liver, 3 lung). Both fecal and tissue samples were stored and transported frozen at -80°C, or were preserved in RNAlater (Sigma-Aldrich Inc., St. Louis, MO, USA), stored initially at -10°C, and finally stored at -80°C until analyses [35, 36].

### RT-PCR, sequencing and phylogenetic analysis

Currently, porcine enteric calicivirus (PEC) Cowden strain [50, 51] is the only known sapovirus that can be cultured. Hence, viral detection and initial characterization involves mostly molecular methods based on sequence data of the well-conserved RNA-dependent RNA polymerase (RdRp) and the variable structural VP1 genes [16, 21]. In this study, sapovirus RNA was detected by targeting the highly conserved RdRp gene.

Total RNA was extracted from 200μl of 10% (wt/vol) fecal suspension in DEPC-treated water using the QIAamp MinElute Virus Spin Kit (QIAGEN, Hilden, Germany), according to manufacturer’s instructions. Sapovirus RNA was detected with the broadly reactive primer pair p289 and p290 [52] targeting highly conserved motifs in the RdRp protein of caliciviruses. Based on the sequences initially generated, nested primers were designed, Cali2F (5’-CAG TGA CAG CCA CAT CCT TG-3’) and Cali2R (5’-AGC ACT GCA GCA GCA AAG TA-3’), targeting the RdRp gene. RT-PCR was performed using SuperScript^™^ III One-Step RT-PCR System with Platinum^®^ Taq DNA Polymerase (Invitrogen, Karlsruhe, Germany) following the user manual’s instructions in a total reaction volume of 25μl. Amplicons of the expected size were purified using the Qiagen PCR purification Kit (Qiagen, Hilden, Germany). In order to avoid RNases, all surfaces were cleansed with RNase away (Molecular BioProducts, San Diego, CA, USA). The purified products were sequenced bidirectional using the Big Dye Terminator Cycle sequencing kit 1.1 (Applied Biosystems [ABI], Darmstadt, Germany) following the manufacturer’s instructions. A 3130 Genetic Analyzer (ABI) was used for the sequencing. Subsequently, sequences were assembled in Geneious v 9.0.2 (Biomatters Ltd, Auckland, New Zealand) or BioEdit 7.0.9.0 [53]. Samples that could not be sequenced were considered positive when bands of the expected size were present with both primer pairs. For these samples the RT-PCRs were run in duplicate to ensure that the results were reliable. To obtain a longer segment of the RdRp gene, the primer 90R (5’-RCC CTC CAT YTC AAA CAC TA-3’) was used together with the primer CaliR2..

GenBank accession numbers for 20 sequences identified by this study are designated KT777545—KT777564. These accession number are included in our phylogenetic tree (Fig 2), together with the host species, the year in which the variant was collected and for spotted hyenas also clan membership, denoted as I,M,P if know or Z if not known. All partial RdRp genes sequences (210 nucleotides, 70 amino-acids) presented the characteristic caliciviral GLPSG motif. One sample from a spotted hyena in 2011 was sequenced for a longer fragment of the RdRp gene (700 nucleotides, 233 amino-acids, accession number KT777560) which presented both the GLPSG motif and the YGDD motif.

Sapovirus sequences obtained in this study for the partial RdRp gene together with others retrieved from Genbank were aligned using the MUSCLE algorithm [54] in Geneious v 9.0.6 (Biomatters Ltd, Auckland, New Zealand). At least one reference sequence of each of the five genogroups of sapovirus (GI–GV) was included in the analysis (GI, n = 5, accession numbers AY237422, AY694184, DQ366345, U95644, U73124, GII, n = 3, AY646855, AY237420, AY603425, GIII, n = 3, FJ715800, AF182760, FJ387164, GIV, n = 1, DQ058829, GV, n = 1, AY646856). Additional sapovirus sequences from domestic dog (JN387135, JN387134), California sea lion (JN420370), mink (AY144337) and bats (JN899072, JN899074, JN899075) were included. Viruses from other genera in the *Caliciviridae* family known to infect carnivores were also included, such as feline calicivirus (AF098931-32) and canine calicivirus (AF053720, AB070225) from the *Vesivirus* genus, and a norovirus reported from a captive African lion (genus *Norovirus*, EF450827). Average nucleotide and amino-acid similarities were calculated using Discovery Studio Visualizer 4.0 (Accelrys Software Inc, San Diego, USA).

Phylogenetic relationships were reconstructed using Maximum Likelihood (ML) and Bayesian Markov chain Monte Carlo (MCMC) phylogenetic inferences. The ML analysis was performed in PAUP* 4.0b10 [55] using 1,000 bootstrap replicates to estimate the statistical support of the branches. The Bayesian analysis was carried out using MrBayes version 3.1 [56, 57]. The MCMC search was set to 10,000,000 iterations, with trees sampled every 1,000^th^ iteration. The nucleotide substitution model used in the ML analysis was obtained using ModelTest 3.7 [58] and for the Bayesian analysis using MrModeltest 2.3 [59]. For both cases the Akaike Information Criterion (AIC) was used to select the best-fitting model.

### Factors influencing sapovirus infection in spotted hyenas

To determine factors influencing the likelihood of sapovirus infection and changes in long-term infection prevalence we screened feces from individually recognized spotted hyenas in three study clans (I,M,P). Age was estimated when individuals were first sighted as cubs, to an accuracy of ± 7 days [60] using pelage characteristics, whether their ears were flattened or upright, and their coordination during locomotion [61, 62]. We classified animals as cubs when less than 12 months of age, as subadults when between 12 and less than 24 months of age, and as adults when ≥ 24 months of age [63]. Sex was determined by the dimorphic glans morphology of the erect phallus [64]. Total clan size comprised all adults, subadults and cubs of both sexes.

Access to food resources in clan territories is determined by social status: all immigrant males are socially subordinate to female clan members and their offspring at food resources in the clan territory [65]. We determined the rank of adults in separate female and breeding male linear dominance hierarchies using the outcome of submissive responses in dyadic interactions within each sex, as detailed in [43, 60, 63]. To compare individual ranks across clans of different sizes, we used standardized ranks. We calculated the standardized rank of each individual within its clan on the date it was sampled using the method described by [66]. This method assigns standardized ranks between -1 (held by the animal with the lowest rank) and +1 (held by the animal with the highest rank) [60, 63]. Adult females with standardized ranks higher or equal to the median standardized rank of 0 were classified as holding high social status, those with standardized ranks below 0 as low social status [43]. Cubs and subadults were assigned the social status of their mother [60]. All immigrant males held a social status below adult clan females [63].

If sapovirus infection depends on intra-specific contact rates, we would expect the dynamics of social interactions within each clan to determine exposure to pathogens. For this purpose we constructed an index of social (ano-genital during greeting ceremonies) contact rates in spotted hyenas as follows. We combined social status and sex in that high ranking females and their offspring were given a high score (for contact rate), low ranking females and their offspring were given a medium score, and immigrant and reproductively active natal males were given a low score. In order to assess whether the range of an animal, the size of the area over which an individual typically roams, determines the chance of exposure to pathogens, we classified adults and subadults of both sexes with an extensive geographical foraging range as ‘roaming’, because they range both within their clan territory (~ 55–75 km^2^) and undertake long distance foraging trips outside the clan territory [46, 47]. Cubs were classified as ‘den-bound’, i.e., with a small range restricted to the vicinity of the communal den inside the clan territory. For the purpose of considering the effect of basic population parameters such as population density on incidence of infection we used total clan size on the date each animal was sampled.

### Incidences of re-infection in spotted hyenas

To investigate whether sapovirus infection provided immunity against re-infections we genetically screened feces from 91 individually known spotted hyenas from which fecal samples were obtained on at least two different dates. Of these, 76 individuals were screened on two different dates, 10 individuals on three different dates and 5 individuals on four different dates. Using these screening results we calculated the average interval duration between two successive sampling dates. We used nonparametric models, including the Mann-Whitney U-test and the Kruskal-Wallis test, to compare medians [67] and the Kaplan-Meier survivorship and the log-rank test in survival analyses to compare the survivorship curves of intervals between different combinations of incidences of infection [68].

### Infection prevalence across years and in age categories

To investigate differences in the prevalence of sapovirus in the spotted hyena population studied between 2001 and 2012 we first tested for differences in the prevalence of infection across years, using a log-likelihood ratio-test. For this test we only considered years with a sample size of at least 20 individuals, thus years 2001, 2002 and 2012 were excluded where sample sizes were 17, 11 and 5, respectively. We also checked for possible differences between age categories, using the same statistical test. These analyses were run in Systat version 13 (Systat Software Inc., Richmond, VA, USA).

### Factors affecting the likelihood of infection in spotted hyenas

We then ran models to assess which of three possible mechanisms influenced the likelihood of sapovirus infection in our study population. For this purpose we used binary logistic regression models [69], with predictor variables contact rate, lifetime range and clan size, and ran these as mixed models with animal identity as a random variable to account for the fact that some individuals contributed more than one tissue or fecal sample to the data set. If a genetic screening result was available for more than one organ or fecal sample for an individual on the same sampling date, only one result was included in the dataset for the prevalence models; if we obtained both a positive and negative result from an animal on the same day, the positive result was selected. This applied to 7 individuals where we had two fecal samples from the same day, and to 7 individuals from which altogether 16 tissue samples were examined. We included data from all individuals sampled during the years which could either be classified as outbreak or non-outbreak years (see Results). Models were run with the glmer function of package lme 4 version 1.1–8 in in R (R Development Core Team, v. 3.1.1).

We used log-likelihood ratio tests and information criteria (AIC and Schwartz’s [BIC_S_] and Raftery’s Bayesian Information Criterion [BIC_R_]) to check whether the final model was superior to an intercept-only or a reduced model. Models were considered similar if differences in AIC were less than 2.5 and preferable if the difference exceeded 6.0 [70]; similar if differences in BIC_R_ were less than 2.0, a positive degree of preference if values of BIC_R_ varied between 2.01 and 6.0 and a strong degree of preference if values of BIC_R_ differed by more than 6 (A. Raftery in [71], p73). As the evaluation of our models with all information criteria produced similar conclusions, we report only AIC values. The significance of each predictor variable was assessed in the following way. We calculated the marginal contribution of each parameter to the full model by subtracting from the full model the log-likelihood ratio of a second model with each variable removed and testing the difference against a chi-square distribution with the appropriate degrees of freedoms (see discussions in [69, 71]).

In order to illustrate the effect of clan size on the chance of infection, we proceeded as follows. We calculated “covariate adjusted estimates” of the logits for each record over the observed range of values by adjusting them to the median of the remaining covariates (contact rate, lifetime range) of their log-odds (logit) for being infected ([69], p80), and then converted the resulting estimates into probabilities using the logistic equation. This permitted us to show the effect of clan size on the likelihood of infection whilst controlling for the covariates contact rate and lifetime range at their middle values. The significance threshold for all tests was fixed at 5% and all tests were two-tailed.

The data used for the statistical analyses is contained in S1 Table.

### Ethics Statement

The study was approved by the Tanzanian Commission of Science and Technology (COSTECH) and the Tanzania Wildlife Research Institute (TAWIRI). Permission to work in the Serengeti National Park was granted by the Tanzanian National Parks Authority (TANAPA). The work was also approved by the Internal Ethics Committee of the Leibniz Institute for Zoo and Wildlife Research (IZW), Approval No. 2011-04-03.

## Results

### Sapovirus detection

Screening targeting the conserved RdRp gene revealed sapoviru*s* RNA in feces from spotted hyena (33.3%, 171/514 samples), African lion (33.3%, 3/9 samples) and bat-eared fox (22.2%, 2/9 samples). No sapoviru*s* RNA was found in fecal samples from golden (0/25) or silver-backed jackals (0/74).

Sapovirus RNA was found in tissue samples collected opportunistically from dead spotted hyenas (spleen, 6/12 samples, liver 1/2 samples, lymph node 2/10 samples), but not in intestine (0/5 samples) or lung samples (0/9 samples). Animals with positive spleen samples were negative for sapovirus RNA in their other available tissues (2 lymph nodes; 1 liver; 1 lung).

### Genetic diversity

A total of 20 partial RdRp gene sequences (16 from spotted hyenas, 3 from African lions and 1 from bat-eared foxes) were obtained and used for the phylogenetic analysis, together with publically available sequence data from 25 representatives of all sapovirus genogroups, divergent unclassified sapoviruses, and other genera in the *Caliciviridae* family, including *Norovirus* and *Vesivirus*. The sapovirus strains from wild carnivore species in the Serengeti ecosystem were placed together in one independent monophyletic cluster (Fig 2), and separately from all recognized sapovirus genogroups (GI to GV) and other unclassified sapoviruses. Nucleotide sequence comparison between strains within the African wild carnivore group and other sapoviruses revealed low nucleotide similarity, ranging from 70.4% ± 1.4 with two domestic dog strains to 56.2% ± 1.5 with sequences from genogroup GII strains. At the amino acid level the highest similarity was with strains within genogroup GIV (84.9% ± 1.5) and the lowest with strains from one bat species in Asia (74.8% ± 0.6). Nucleotide sequence comparison with members of other genera in the *Calicivirus* family known to infect carnivores also showed low similarity values (feline and canine calicivirus; 60.3% ± 1.7 and 59.3% ± 2.1, respectively, norovirus from a captive African lion; 56.1% ± 0.9).

Within the group of African wild carnivore strains, the sapovirus strains from spotted hyenas grouped together and separately from those obtained from African lions and bat-eared foxes (Fig 2). One strain from a member of the P clan in 2007 was placed separately from all other strains obtained from this species between 2002 and 2011. Two other variants from spotted hyenas in 2007 were placed together with strains obtained in 2002 and 2006 from this species (Fig 2). The other three major groups include two clusters that contained strains obtained in two consecutive years (from 2010 and 2011 and from 2004 and 2005), and another cluster containing strains obtained in 2002, 2003, 2004, and 2006. With the exception of the cluster containing variants from 2004 and 2005 (all from members of the P study clan), all the clusters contained variants obtained from members of at least two of the three study clans. Notably, one variant obtained from a bat-eared fox in 2011 was placed separately from those obtained from spotted hyenas in that same year, but close to the three variants obtained in 2010 from African lions.

Comparison of nucleotide sequences revealed that the average similarity between all strains from spotted hyenas (96.1% ± 3.8) was lower than between strains from African lions and bat-eared foxes (99.1% ± 0.3). The percentage similarity from the variant in 2007 placed separately from the other spotted hyena variants was lower (87.4% ± 0.6) than that of all the other spotted hyena variants that grouped more closely (97.4% ± 1.6). However, comparison at the amino acid level revealed that all strains from spotted hyenas were identical. The average similarity of the sequences from African lions and bat-eared foxes was 99.2% ± 0.9 (with differences at two amino acid positions). The average nucleotide and amino acid similarity between the spotted hyena strains and those from African lions and bat-eared foxes was 85.1% ± 1.0 and 99.6% ± 0.7, respectively.

### Sapovirus infection prevalence in spotted hyenas

**Fig 3:**
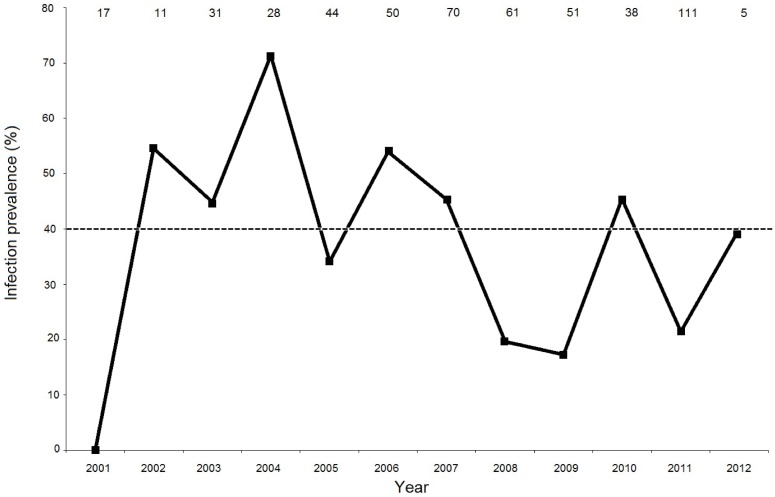
Prevalence of sapovirus infection in spotted hyenas in the Serengeti National Park. Numbers at the top of the plot indicate sample sizes for each year from 2001 to 2012.

Overall, sapovirus infection prevalence (combining results from fecal and tissue samples) in spotted hyenas was 34.8% (180/517 samples, Table 1). Infection prevalence between 2001 and 2012 (Fig 3) fluctuated substantially between years (log likelihood ratio = 69.157, df = 11, p < 0.00001). We considered infection prevalence in any given year equal to or above 40% as indicative of an outbreak of sapovirus infection in that year. By this definition, 2003, 2004, 2006, 2007, 2010 were considered ‘outbreak years’, and 2005, 2008, 2009, 2011 were considered ‘non-outbreak years’ in which infection prevalence was below 40%. Of the 16 partial RdRp gene sequences obtained from spotted hyenas, 11 of these were from outbreak years, three were from non-outbreak years and two were from years that could not be classified (2002). In the phylogenetic tree strains from non-outbreak years clustered with those from outbreak years (Fig 2).

**Table 1:** Sapovirus infection prevalence in spotted hyenas during outbreak years and non-outbreak years.

Age category	Non-outbreak years	Outbreak years	All years*
Adults	19 / 59; **32.2**%	36 / 73; **49.3**%	56 / 146; **38.4**%
Subadults	5 / 20; **25.0**%	5 / 16; **31.3**%	11 / 41; **26.8**%
Cubs	37 / 188; **19.7**%	70 / 128; **54.7**%	113 / 330; **34.2**%
Total	61 / 267; **22.8**%	111 / 217; **51.2**%	180 / 517; **34.8**%

Sapovirus RNA positive samples / total sample size and infection prevalences (%) are shown for spotted hyenas in different age categories.

* all years: includes also data from the years which could not be classified as outbreak or non-outbreak years, hence sample sizes are larger than the sums from non-outbreak and outbreak years.

To determine whether the prevalence of sapovirus infection was affected by age, we screened feces from animals in different age categories (i.e., cubs, subadults and adults). We found no significant differences in infection prevalence between different age categories across all years (chi square test, likelihood ratio = 2.045, df = 2, p = 0.36), in non-outbreak years (likelihood ratio = 3.860, df = 2, p = 0.15), or outbreak years (likelihood ratio = 3.331, df = 2, p = 0.19).

### Incidence of re-infection in spotted hyenas

We screened for sapovirus RNA in feces obtained from 91 individuals on two separate occasions (S2 Table). Of these, 15 individuals were sampled on at least three separate occasions, and from five of these animals on a fourth occasion (Table 2). Results revealed 32 transitions of an individual from sapovirus RNA negative to positive and 15 transitions from positive to negative. In many cases the infection status did not change between sampling dates, for both initially negative (negative to negative, 53 cases) and initially positive individuals (positive to positive, 11 cases). We found no cases of transitions from positive to negative to positive (Table 2).

**Table 2:** RT-PCR fecal screening results for known spotted hyenas sampled at least three dates.

Animal	First sample	Time interval (days)	Second sample	Time interval (days)	Third sample	Time interval (days)	Fourth sample
A	Neg	124	Neg	10	Neg		
B	Neg	19	Neg	82	Neg	188	
C	Neg	23	Neg	131	Neg	563	Neg
D	Neg	26	Neg	1698	Pos		
E	Neg	9	Neg	969	Pos		
F	Neg	35	Neg	110	Pos		
G	Neg	20	Neg	5	Pos		
H	Neg	16	Neg	20	Neg	352	Pos
I	Neg	6	Neg	11	Neg	22	Pos
J	Neg	118	Pos	715	Pos		
K	Pos	755	Pos	5	Neg		
L	Pos	3	Neg	99	Neg		
M	Pos	16	Neg	29	Neg		
N	Pos	143	Neg	38	Neg		
O	Pos	27	Neg	35	Neg	41	Neg

Sapovirus RNA infection status: positive (Pos), negative (Neg) for 15 known spotted hyenas (animals A-O) sampled on at least three separate dates and the time interval in days between sampling dates.

Transition intervals were similar between first and second, second and third, and third and fourth sampling date (Kruskal-Wallis test, H = 2.567, df = 2, p = 0.28). We therefore analyzed the relationship between the duration of time intervals between successive samples and changes in infection status without regard to the number sampling repeats per individual. The duration significantly varied between different categories of changes of infection status (survival analysis, log-rank test, log-likelihood ratio = 10.114, df = 3, p = 0.018). Similar intervals were observed for changes in infection status from negative to positive (mean: 466.6 days, 95% C.I. 285.5–647.6 days, median 158 days, n = 32) and from positive to negative (mean: 602.8 days, 95% C.I. 185.8–1019.8 days, median 180 days, n = 15). The shortest and longest intervals were observed when there was no change in status: negative to negative (mean: 241.1 days, 95% C.I. 132.5–349.7 days, median 82 days, n = 53) and positive to positive (mean: 769.0 days, 95% C.I. 412.5–1125.5 days, median 715 days, n = 11), respectively.

### Factors influencing the likelihood of sapovirus infection in spotted hyenas

**Fig 4:**
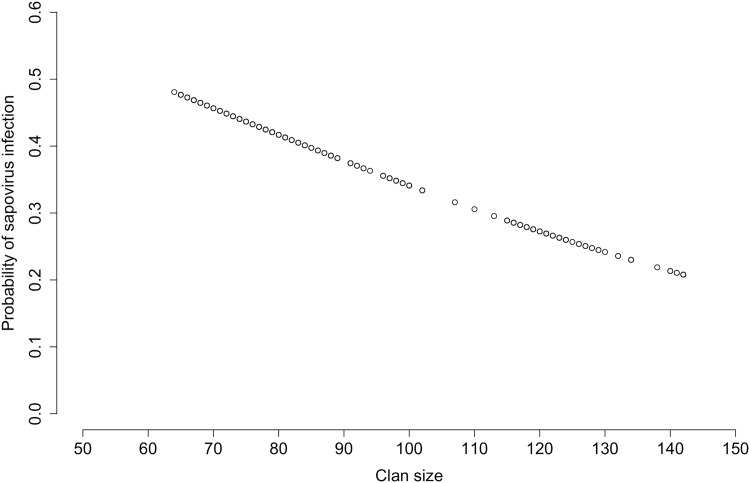
The likelihood of infection of spotted hyenas with sapovirus as a function of clan size. Note that the probabilities were adjusted for the potential effects of the other covariates, contact rate and lifetime range, by setting them to the median of their values (for details see Material and Methods). Circles represent predicted values within the range of actual clan sizes observed.

We used a mixed-effects binary logistic regression model to test factors influencing the likelihood of infection in spotted hyenas (log-likelihood ratio = 16.717, df = 4, p = 0.0022, n = 484 samples from 380 individuals with complete information). The results revealed that infection was not significantly altered by either contact rates or the extent of an individual’s range (Table 3). The likelihood of infection significantly declined as clan size increased: with every additional individual in the clan, clan members were 1.02 times less likely to be infected with sapovirus (Table 3). As the actual clan sizes ranged from 65 to 145 individuals, this implied a more than two-fold change in the likelihood of infection across the observed range of clan sizes (Fig 4). Correspondingly, median clan sizes were significantly lower during outbreak (median = 77, mean = 83.6, with 95% C.I.: 81.4–85.9) than non-outbreak (median = 89, mean = 99.2, with 95% C.I.: 96.3–102.0) years (Mann-Whitney U-test, U = 41981.5, N_outbreak_ = 217, N_non-outbreak_ = 267, p < 0.00001).

**Table 3:** The effect of social contact rate, individual lifetime range and clan size on the likelihood of sapovirus infection in spotted hyenas.

	Regression coefficients								Odds ratios			AIC	
	β	SE	z	p	G	p	95% CI		Estimate	95% CI		AIC_r_	Δ AIC
							Lower	Upper		Lower	Upper		
(Intercept)	1.066	0.458	2.328	0.02			0.168	1.963	2.903	1.183	7.122	633.8*	8.72
Contact rate (medium ≤ high)	-0.282	0.205	-1.374	0.17	2.032	0.36	-0.684	0.120	0.754	0.505	1.128	623.2	-1.97
Contact rate (low ≤ high)	-0.008	0.366	-0.021	0.98			-0.726	0.710	0.992	0.484	2.035		
Range (den-bound ≤ roaming)	-0.109	0.227	-0.480	0.63	0.23	0.63	-0.553	0.335	0.897	0.575	1.399	623.4	-1.77
Clan size	-0.016	0.0046	-3.481	0.0005	12.773	0.0004	-0.0253	-0.007	0.984	0.975	0.993	635.9	10.77

Shown are the estimates of the regression coefficients from a mixed-effects binary logistic regression model (n = 484 samples from 380 individuals) with their standard errors (SE) and 95% confidence limits in natural log-units as well as their conversion into odds ratios with their respective 95% confidence limits. Positive (negative) estimates indicate that an increase in the value of the parameter increased (reduced) the incidence of infection. Also shown are the tests for the significance of each parameter using log-likelihood ratio tests, except for the *intercept where it is the z-value and its associated p-value based on the estimate and its SE. Also shown are the values for the Akaike Information Criterion (AIC_r_) for each reduced model when the specific predictor was removed and the difference Δ AIC to the full model. The AIC for the full model was 625.2.

* null (intercept only) model

## Discussion

This is the first report of sapovirus infection in wildlife species in Africa. Our results extend the host species range for this genus to include the spotted hyena, African lion and bat-eared fox. Prior to our study, sapovirus infection in carnivores worldwide was not known from any species belonging to the Felidae (including the domestic cat) [72], or Hyaenidae, but was reported only for species in the families Otariidae (Californian sea lion) [23], Mustelidae (mink) [25] and Canidae (domestic dog) [26].

Our phylogenetic analysis based on partial RdRp gene sequences revealed that sapovirus strains from wild carnivores in the Serengeti ecosystem formed a monophyletic group that was distinct from other sapovirus strains worldwide, including strains from the three previously identified carnivore hosts (Fig 2). Strains from spotted hyena formed a separate sub-group from those obtained from African lions and bat-eared foxes, even within the same sampling year (Fig 2), suggesting that strains circulating in the spotted hyena population are distinct from those in the African lion and bat-eared fox populations. Evidence for a degree of species-specificity in host range is apparent in other viruses of carnivores in the Serengeti ecosystem. Genetically distinct alphacoronavirus variants infect spotted hyenas and sympatric silver-backed jackal during the same year [35], genetically distinct strains of kobuvirus infect domestic dogs and wild carnivores [36], and during the 1993/1994 canine distemper epidemic in the Serengeti NP, genetically distinct strains circulated in non-canids (African lion and spotted hyena) and canids (domestic dog and bat-eared fox) [73]. More extensive characterization of sapovirus strains infecting carnivore species in the Serengeti ecosystem would clarify their host range and help identify which species in the large carnivore guild are infected with sapovirus. Currently it is not known whether or not domestic dogs and domestic cats in Africa are infected with sapovirus.

Our results support the conclusion of previous studies, which emphasize the importance of long-term monitoring when documenting the genetic diversity of sapovirus strains [20, 28, 31]. Clearly we would have detected far less genetic diversity in our partial RdRp gene sequence data had our sampling of spotted hyenas been limited to a time frame of one or two years (Fig 2), and particularly if sampling was (by chance) only undertaken during non-outbreak years when infection prevalence was low (Fig 3). Samples obtained during outbreak years revealed considerable genetic diversity; for example from 2006 to 2007 we obtained sequence data from five different variants, including the distinct 2007 variant (KT777556) which was the least similar to all others from this host species. As we were not able to sequence data from all RT-PCR positive samples, we cannot exclude the possibility that the genetic diversity among spotted hyena strains was higher than our results indicate. Even so, in line with a previous study [74], our result show that sequence data from the non-structural RdRp gene yields useful information on the genetic diversity of circulating sapovirus strains.

Some outbreaks of sapovirus infection in humans can be linked to the emergence of specific genotypes [18, 75, 76], suggesting that herd immunity against prevailing genotypes may be evaded by the emergence of genetically novel strains. Our long-term monitoring of sapovirus infection in spotted hyenas revealed significant changes in yearly prevalence during the study (Fig 3) and the occurrence of three outbreaks of infection. The highest infection prevalence (above 72.4%) occurred during an outbreak from 2002 to 2004, whereas infection prevalence in two later outbreaks (from 2006 to 2007 and in 2010) was considerably lower. Presumably the 2002/2004 sapovirus outbreak induced herd immunity to the genetic strains that circulated in spotted hyenas during this period, but our phylogenetic analysis (Fig 2) did not reveal the emergence of genetically distinct strains in response to this. Even so, our partial sapovirus RdRp gene sequence data are insufficient to draw strong conclusions. For this, a more extensive genetic investigation is needed, particularly using the VP1 gene used to place sapoviruses in genogroups [16, 21], that may reflect the antigenic relationships between sapovirus [77]. Overall sapovirus infection prevalence in spotted hyenas (34.8%) in the Serengeti NP was several magnitudes higher than the prevalence reported for the domestic dog (< 2%) [26, 72] and the bat *H*. *pomona* (1.6%) [28]. Moreover, our long-term monitoring reveals that infection prevalence in spotted hyenas was typically high, being above 20% in most years (Fig 3).

There has been much discussion about the effect of human age on sapovirus infection (reviewed by [16]), mostly based on studies on individuals with gastrointestinal infections. However, there is growing evidence from research on humans with and without clinical symptoms which demonstrates sapovirus infection across a wide range of ages [76], including elderly people [78]. Our results on sapovirus infection across different age categories indicate that the likelihood of infections in spotted hyenas was not significantly influenced by age (Table 1).

The long-term perspective of our study allowed us to assess the sapovirus infection status of several individually known spotted hyenas on different sampling dates. Several animals transitioned from positive to negative, and we interpret this to indicate that they successfully cleared the virus following infection. If, following an initial infection, spotted hyenas gained long-term immunity against further infection, infection prevalence should decline with age. As prevalence amongst adults reached almost 50% during outbreak years, our results do not provide strong evidence for long-term immunity in this species (Table 1). Even so, we found no individual that changed from RT-PCR positive to negative to positive (Table 2) which would have provided direct evidence of re-infection. During outbreaks of sapovirus in humans and pigs, cases of re-infection with sapovirus belonging to different genogroups have been reported in both species, suggesting genogroup-specific immunity for sapoviruses [74, 79, 80]. More extensive investigation of the genetic diversity across strains circulating in our spotted hyena population is needed to determine whether sapovirus strains induce (1) short-term immunity, which would permit re-infection with strains from the same sapovirus genogroup, (2) genogroup-specific immunity, in which re-infection would involve strains from different genogroups, or (3) possibly a complex interplay between the two, as hypothesized for the genetically and antigenically diverse *Norovirus* which is closely related to the *Sapovirus* genus[81, 82].

Interestingly, infection status depended on the length of time between repeated samples. Animals that were negative on two separate dates (Table 2) had the shortest median period between sampling dates (82 days), those that changed from negative to positive (158 days) and from positive to negative (180 days) had an intermediate median number of days, whereas animals that were positive on both sampling days (715 days) had the longest median period. Taken together, these results suggest that when a spotted hyena is infected, infection is cleared, and reinfection is unlikely within a period of several months, which is consistent with the idea that exposure to sapoviru*s* does not provide long-term immunity against further infection. In humans, sapovirus shedding often subsided 14 days after the onset of illness [83], but can persist for up to 38 days [74]. Hence, we speculate that the spotted hyenas which were positive on two sampling dates several months apart were animals that were re-infected rather than individuals with persistent long-term infections. However, currently we cannot exclude the possibility that there may be spotted hyenas that shed sapoviruses for periods spanning several months.

Spotted hyena social and ranging behavior has been shown to structure transmission routes and the likelihood of infection by a broad range of pathogens [35, 41–44]. Yet when we tested whether these two factors influenced sapovirus infection in this species, neither the predicted effect of contact rates based on known patterns of greeting ceremonies, nor the extent of an individual’s range significantly influenced the likelihood of infection in our study population (Table 3). Clan size was a significant factor, but contrary to our expectation, the likelihood of infection declined with increasing clan size. This phenomenon is known in the behavioral literature as the attack-abatement effect [84] or as the encounter-reduction effect [48, 85, 86]. In multi-species host assemblages the same phenomenon in the ecological literature is termed a ‘dilution’ effect. At least five non-exclusive mechanisms have been identified [49] that can cause a reduction in infection incidence as the number of host species increases, but not all of them are relevant to intraspecific sapovirus infections in spotted hyenas. We have no evidence that sapovirus infection increases the death rate of infected individuals (mechanism 1), as no obvious clinical signs are associated with sapovirus infection in most spotted hyenas. Although we lack a precise measure of the recovery rate (mechanism 2), the general absence of obvious clinical signs of sapovirus infection suggest that the recovery rate is already very high, so that a change in this factor is likely to be modest. A decrease in the density of susceptible individuals (mechanism 3) is unlikely unless this results from a substantial increase in the proportion of clan members immune to sapovirus infection, even if immunity is relatively transient. A substantial increase in the prevalence of immune clan members would result in far fewer sapovirus susceptible individuals. Moreover, such an increase in herd immunity would probably also lead to a decrease in the prevalence of sapovirus excreting clan members, thereby reducing the probability of: (1) sapovirus transmission per encounter (mechanism 4) between clan members and (2) the encounter rate between susceptible and infectious individuals (mechanism 5) in a clan. Further research and more detailed modeling of the interplay between clan size and the prevalence of clan members in different sapovirus infection states (susceptible, infected/excreting virus, and immune) is required to test this idea.

In the context of our study, mechanism 5 (encounter rate between susceptible and infectious individuals) is similar to mechanism 3 (decrease in the density of susceptible individuals) because a reduced density of susceptible clan members caused by a rise in the prevalence of transient immunity would also curb the number of infected animals in a clan and may prevent their number growing in proportion to increasing clan size, and possibly holding them at or below a fixed number (threshold). An increase in the risk of internal pathogen infection with a decrease in a group size component has been reported by other studies [3, 42].

As sapoviruses cannot be cultured, with the exception of the strain PEC Cowden [50, 51], knowledge of viral tropism and the receptor use for entry to host cells is limited. Moreover, sapovirus typically infects intestinal tissue and to our knowledge is not known to infect other tissues, as illustrated by the absence of viral RNA in any extra-intestinal tissues in gnotobiotic pigs inoculated with PEC Cowden [87]. However, in a few dead spotted hyenas (typically road casualties) from which we opportunistically obtained tissue samples, we detected sapovirus RNA most often in the spleen and occasionally also in lymph nodes, intestines and the liver. To our knowledge this is the first report of sapovirus RNA in extra-intestinal tissues following natural infections. The possibility that sapoviruses disseminate to extra-intestinal tissues may be of clinical importance [88]. Notably, in asymptomatic mice shedding murine norovirus in feces, viral RNA was also found in several extra-intestinal organs, including the liver, spleen and lymph nodes [89].

Studies on caliciviruses infecting wild carnivores have focused on feline calicivirus (FCV, genus *Vesivirus*, family *Caliciviridae*). For example, serological surveys documented that African lions [90] and spotted hyenas in the Serengeti ecosystem were exposed to FCV [91] with a high prevalence. Our phylogenetic analysis shows that the variants we report from wild carnivores in Africa are distinct from both FCV and canine calicivirus (Fig 2). A norovirus (genus *Norovirus*, family *Caliciviridae*), genetically related to human noroviruses was reported from a captive African lion [92] and subsequently identified in domestic dogs and domestic cats [93, 94]. Combining these findings with our results suggests that the spotted hyena and African lion can be infected by viruses belonging to three different genera of the family *Caliciviridae*.

We provide the first report of sapovirus infection in wild carnivore species in Africa, including the spotted hyena, African lion and bat-eared fox. Long-term monitoring revealed considerable genetic diversity of variants from these species which were phylogenetically distinct from previously reported sapovirus strains from other geographical areas worldwide. Sapovirus prevalence in the spotted hyenas varied between years and was not influenced by age. An individual’s likelihood of infection significantly declined with increasing group size, consistent with an encounter reduction effect. Our results reinforce the importance of long-term studies in viral epidemiology of wild species.

## Supporting Information

S1 Table Data set for the mixed-effects binary logistic regression model. (PDF)

S2 Table Data set for the analysis of repeatedly sampled spotted hyenas. (PDF)
