# Supplementary material for: Divergent Sapovirus Strains and Infection Prevalence in Wild Carnivores in the Serengeti Ecosystem: A Long-Term Study
Source: PLoS One. 2016 Sep 23;11(9):e0163548. doi: 10.1371/journal.pone.0163548 (PMC5035092; doi:10.1371/journal.pone.0163548)
Supplement: S1 Table — (PDF) [file pone.0163548.s001.pdf]

Supporting Information

Divergent sapovirus strains and infection prevalence in wild carnivores in the Serengeti ecosystem: a long-term study

Ximena A Olarte-Castillo, Heribert Hofer, Katja V Goller, Vito Martella, Patricia D Moehlman, Marion L East

S1 Table: Data set for the binary logistic regression model.

Year	Outbreak	Samp_Type	Contact	Roamer	Ad_Juv	Clan_Size	Sapovirus_Pcr
2003	outbreak	faeces	2_M	2_den-bound	cub	69	0
2003	outbreak	faeces	1_H	1_roaming	ad	81	0
2003	outbreak	faeces	2_M	2_den-bound	cub	65	0
2003	outbreak	faeces	1_H	2_den-bound	cub	65	0
2003	outbreak	faeces	2_M	1_roaming	ad	65	0
2003	outbreak	faeces	2_M	2_den-bound	cub	65	0
2003	outbreak	faeces	2_M	2_den-bound	cub	65	0
2003	outbreak	faeces	2_M	2_den-bound	cub	65	0
2003	outbreak	faeces	2_M	2_den-bound	cub	67	1
2003	outbreak	faeces	2_M	2_den-bound	cub	68	1
2003	outbreak	spleen	1_H	2_den-bound	cub	81	0
2003	outbreak	lung	2_M	1_roaming	subad	81	0
2003	outbreak	faeces	1_H	1_roaming	subad	64	0
2003	outbreak	faeces	1_H	2_den-bound	cub	75	1
2003	outbreak	faeces	2_M	1_roaming	subad	75	1
2003	outbreak	faeces	1_H	2_den-bound	cub	65	1
2003	outbreak	faeces	1_H	1_roaming	subad	65	1
2003	outbreak	faeces	2_M	1_roaming	ad	71	0
2003	outbreak	faeces	2_M	2_den-bound	cub	71	1
2003	outbreak	faeces	2_M	2_den-bound	cub	71	1
2003	outbreak	faeces	2_M	2_den-bound	cub	65	1
2003	outbreak	faeces	3_L	1_roaming	ad	72	1
2003	outbreak	faeces	2_M	2_den-bound	cub	69	0
2003	outbreak	faeces	1_H	1_roaming	ad	74	0
2003	outbreak	faeces	2_M	1_roaming	ad	71	0
2003	outbreak	faeces	2_M	1_roaming	ad	76	0
2003	outbreak	faeces	1_H	2_den-bound	cub	76	0
2003	outbreak	intest	1_H	2_den-bound	cub	67	1
2003	outbreak	faeces	1_H	1_roaming	ad	67	1

2003	outbreak	faeces	1_H	2_den-bound	cub	67	1
2003	outbreak	faeces	1_H	2_den-bound	cub	67	1
2004	outbreak	faeces	1_H	2_den-bound	cub	66	1
2004	outbreak	faeces	1_H	2_den-bound	cub	67	1
2004	outbreak	faeces	2_M	1_roaming	ad	75	0
2004	outbreak	faeces	3_L	1_roaming	ad	67	0
2004	outbreak	faeces	1_H	2_den-bound	cub	66	1
2004	outbreak	faeces	2_M	1_roaming	subad	66	1
2004	outbreak	faeces	1_H	2_den-bound	cub	66	1
2004	outbreak	faeces	2_M	2_den-bound	cub	66	1
2004	outbreak	faeces	1_H	2_den-bound	cub	78	1
2004	outbreak	faeces	2_M	2_den-bound	cub	73	1
2004	outbreak	faeces	3_L	1_roaming	ad	73	1
2004	outbreak	faeces	2_M	2_den-bound	cub	73	0
2004	outbreak	faeces	1_H	2_den-bound	cub	67	1
2004	outbreak	faeces	3_L	1_roaming	ad	71	0
2004	outbreak	faeces	1_H	2_den-bound	cub	71	1
2004	outbreak	faeces	2_M	1_roaming	ad	98	1
2004	outbreak	faeces	1_H	2_den-bound	cub	73	1
2004	outbreak	faeces	2_M	2_den-bound	cub	73	1
2004	outbreak	faeces	2_M	1_roaming	ad	71	0
2004	outbreak	faeces	1_H	2_den-bound	cub	73	0
2004	outbreak	faeces	2_M	1_roaming	ad	71	0
2004	outbreak	faeces	1_H	2_den-bound	cub	74	1
2004	outbreak	faeces	2_M	2_den-bound	cub	67	1
2004	outbreak	faeces	2_M	2_den-bound	cub	74	1
2004	outbreak	lymphNode	2_M	2_den-bound	cub	74	1
2004	outbreak	faeces	2_M	2_den-bound	cub	68	0
2004	outbreak	faeces	1_H	2_den-bound	cub	68	1
2004	outbreak	faeces	2_M	2_den-bound	cub	69	1
2005	non-outbreak	faeces	1_H	2_den-bound	cub	79	1
2005	non-outbreak	faeces	2_M	2_den-bound	cub	70	0
2005	non-outbreak	faeces	1_H	2_den-bound	cub	69	0
2005	non-outbreak	faeces	2_M	2_den-bound	cub	69	0
2005	non-outbreak	faeces	2_M	2_den-bound	cub	69	0
2005	non-outbreak	faeces	1_H	1_roaming	ad	66	0
2005	non-outbreak	faeces	1_H	2_den-bound	cub	71	1
2005	non-outbreak	faeces	3_L	1_roaming	ad	66	0
2005	non-outbreak	faeces	2_M	2_den-bound	cub	66	0
2005	non-outbreak	faeces	1_H	1_roaming	subad	66	0
2005	non-outbreak	faeces	2_M	1_roaming	ad	74	1
2005	non-outbreak	faeces	3_L	1_roaming	ad	74	0
2005	non-outbreak	faeces	1_H	2_den-bound	cub	74	1
2005	non-outbreak	faeces	1_H	1_roaming	ad	75	0
2005	non-outbreak	faeces	1_H	2_den-bound	cub	74	0
2005	non-outbreak	faeces	1_H	1_roaming	ad	79	1
2005	non-outbreak	faeces	2_M	1_roaming	subad	68	0
2005	non-outbreak	faeces	2_M	2_den-bound	cub	79	1
2005	non-outbreak	faeces	2_M	2_den-bound	cub	79	1

2005	non-outbreak	faeces	2_M	2_den-bound	cub	79	1
2005	non-outbreak	faeces	2_M	2_den-bound	cub	79	0
2005	non-outbreak	faeces	3_L	1_roaming	ad	68	0
2005	non-outbreak	faeces	2_M	2_den-bound	cub	67	1
2005	non-outbreak	faeces	1_H	1_roaming	ad	67	0
2005	non-outbreak	faeces	1_H	1_roaming	ad	72	0
2005	non-outbreak	intest	1_H	2_den-bound	cub	67	0
2005	non-outbreak	faeces	1_H	1_roaming	ad	66	0
2005	non-outbreak	faeces	1_H	1_roaming	ad	71	1
2005	non-outbreak	faeces	1_H	2_den-bound	cub	74	0
2005	non-outbreak	lung	1_H	2_den-bound	cub	74	0
2005	non-outbreak	faeces	1_H	2_den-bound	cub	89	0
2005	non-outbreak	faeces	2_M	2_den-bound	cub	74	0
2005	non-outbreak	faeces	1_H	2_den-bound	cub	74	0
2005	non-outbreak	faeces	3_L	1_roaming	ad	69	1
2005	non-outbreak	faeces	2_M	1_roaming	ad	69	0
2005	non-outbreak	faeces	3_L	1_roaming	ad	69	1
2005	non-outbreak	faeces	2_M	2_den-bound	cub	69	0
2005	non-outbreak	faeces	1_H	1_roaming	subad	89	0
2005	non-outbreak	faeces	1_H	2_den-bound	cub	89	0
2005	non-outbreak	faeces	1_H	2_den-bound	cub	89	1
2005	non-outbreak	faeces	1_H	1_roaming	ad	67	1
2005	non-outbreak	faeces	2_M	1_roaming	subad	67	0
2005	non-outbreak	faeces	2_M	1_roaming	ad	69	0
2005	non-outbreak	faeces	3_L	1_roaming	ad	69	1
2006	outbreak	faeces	1_H	2_den-bound	cub	74	0
2006	outbreak	faeces	1_H	2_den-bound	cub	74	1
2006	outbreak	faeces	2_M	2_den-bound	cub	89	1
2006	outbreak	faeces	3_L	1_roaming	ad	72	1
2006	outbreak	faeces	3_L	1_roaming	ad	72	1
2006	outbreak	faeces	1_H	2_den-bound	cub	74	0
2006	outbreak	faeces	1_H	2_den-bound	cub	74	1
2006	outbreak	faeces	1_H	2_den-bound	cub	72	1
2006	outbreak	faeces	1_H	1_roaming	ad	74	1
2006	outbreak	faeces	1_H	2_den-bound	cub	88	1
2006	outbreak	faeces	2_M	2_den-bound	cub	74	0
2006	outbreak	faeces	3_L	1_roaming	ad	88	0
2006	outbreak	faeces	3_L	1_roaming	ad	88	0
2006	outbreak	faeces	1_H	1_roaming	ad	72	1
2006	outbreak	faeces	2_M	1_roaming	ad	74	0
2006	outbreak	faeces	2_M	1_roaming	subad	74	0
2006	outbreak	faeces	2_M	2_den-bound	cub	72	0
2006	outbreak	faeces	1_H	2_den-bound	cub	72	1
2006	outbreak	faeces	2_M	1_roaming	ad	88	0
2006	outbreak	faeces	2_M	2_den-bound	cub	88	1
2006	outbreak	faeces	2_M	2_den-bound	cub	72	1
2006	outbreak	faeces	2_M	2_den-bound	cub	73	0
2006	outbreak	faeces	2_M	2_den-bound	cub	73	0
2006	outbreak	faeces	2_M	2_den-bound	cub	73	0

2006	outbreak	faeces	1_H	1_roaming	subad	88	0
2006	outbreak	faeces	1_H	2_den-bound	cub	88	1
2006	outbreak	faeces	3_L	1_roaming	ad	75	1
2006	outbreak	faeces	1_H	2_den-bound	cub	74	0
2006	outbreak	faeces	2_M	2_den-bound	cub	74	0
2006	outbreak	faeces	1_H	2_den-bound	cub	91	1
2006	outbreak	faeces	1_H	2_den-bound	cub	91	1
2006	outbreak	faeces	1_H	1_roaming	ad	73	1
2006	outbreak	faeces	3_L	1_roaming	ad	81	1
2006	outbreak	faeces	2_M	2_den-bound	cub	79	0
2006	outbreak	faeces	1_H	1_roaming	ad	94	0
2006	outbreak	faeces	2_M	2_den-bound	cub	81	1
2006	outbreak	faeces	2_M	2_den-bound	cub	81	0
2006	outbreak	faeces	2_M	2_den-bound	cub	93	1
2006	outbreak	faeces	1_H	2_den-bound	cub	76	1
2006	outbreak	faeces	1_H	2_den-bound	cub	78	1
2006	outbreak	faeces	2_M	2_den-bound	cub	78	1
2006	outbreak	faeces	1_H	2_den-bound	cub	78	1
2006	outbreak	faeces	3_L	1_roaming	ad	78	0
2006	outbreak	faeces	1_H	2_den-bound	cub	78	1
2006	outbreak	faeces	1_H	2_den-bound	cub	78	0
2006	outbreak	faeces	1_H	2_den-bound	cub	78	0
2006	outbreak	faeces	1_H	1_roaming	ad	79	1
2006	outbreak	faeces	1_H	2_den-bound	cub	79	0
2006	outbreak	faeces	3_L	1_roaming	ad	97	0
2006	outbreak	faeces	1_H	2_den-bound	cub	78	1
2007	outbreak	faeces	3_L	1_roaming	ad	70	1
2007	outbreak	faeces	2_M	1_roaming	ad	70	0
2007	outbreak	faeces	3_L	1_roaming	ad	70	1
2007	outbreak	faeces	1_H	2_den-bound	cub	70	0
2007	outbreak	faeces	1_H	2_den-bound	cub	69	0
2007	outbreak	faeces	1_H	2_den-bound	cub	71	0
2007	outbreak	faeces	3_L	1_roaming	ad	70	0
2007	outbreak	faeces	3_L	1_roaming	ad	100	1
2007	outbreak	faeces	2_M	2_den-bound	cub	100	0
2007	outbreak	faeces	3_L	1_roaming	ad	77	0
2007	outbreak	faeces	2_M	2_den-bound	cub	100	0
2007	outbreak	faeces	2_M	2_den-bound	cub	100	0
2007	outbreak	faeces	2_M	1_roaming	subad	77	0
2007	outbreak	faeces	3_L	1_roaming	ad	77	0
2007	outbreak	faeces	3_L	1_roaming	ad	76	1
2007	outbreak	faeces	1_H	1_roaming	ad	97	1
2007	outbreak	faeces	1_H	1_roaming	ad	75	1
2007	outbreak	faeces	1_H	2_den-bound	cub	75	1
2007	outbreak	faeces	1_H	1_roaming	ad	76	0
2007	outbreak	faeces	2_M	2_den-bound	cub	78	1
2007	outbreak	faeces	1_H	2_den-bound	cub	78	1
2007	outbreak	faeces	1_H	2_den-bound	cub	97	0
2007	outbreak	faeces	1_H	2_den-bound	cub	78	1

2007	outbreak	faeces	2_M	2_den-bound	cub	78	0
2007	outbreak	faeces	1_H	2_den-bound	cub	78	1
2007	outbreak	faeces	3_L	1_roaming	ad	78	0
2007	outbreak	faeces	2_M	1_roaming	ad	77	0
2007	outbreak	faeces	1_H	1_roaming	ad	100	0
2007	outbreak	faeces	1_H	2_den-bound	cub	100	0
2007	outbreak	faeces	1_H	1_roaming	ad	100	0
2007	outbreak	faeces	2_M	1_roaming	subad	100	0
2007	outbreak	faeces	2_M	1_roaming	subad	100	0
2007	outbreak	faeces	2_M	2_den-bound	cub	78	1
2007	outbreak	faeces	1_H	2_den-bound	cub	78	1
2007	outbreak	faeces	2_M	2_den-bound	cub	74	0
2007	outbreak	faeces	1_H	2_den-bound	cub	99	0
2007	outbreak	faeces	2_M	1_roaming	ad	75	1
2007	outbreak	faeces	1_H	2_den-bound	cub	97	1
2007	outbreak	faeces	1_H	2_den-bound	cub	97	0
2007	outbreak	faeces	1_H	1_roaming	ad	78	1
2007	outbreak	faeces	1_H	1_roaming	ad	78	0
2007	outbreak	faeces	1_H	1_roaming	ad	75	1
2007	outbreak	faeces	1_H	1_roaming	subad	75	0
2007	outbreak	faeces	1_H	1_roaming	subad	75	1
2007	outbreak	faeces	2_M	1_roaming	ad	98	1
2007	outbreak	faeces	1_H	1_roaming	ad	74	1
2007	outbreak	faeces	2_M	2_den-bound	cub	74	1
2007	outbreak	faeces	2_M	1_roaming	ad	97	1
2007	outbreak	faeces	1_H	1_roaming	ad	73	1
2007	outbreak	faeces	2_M	1_roaming	ad	97	1
2007	outbreak	faeces	1_H	1_roaming	ad	73	1
2007	outbreak	faeces	1_H	1_roaming	ad	73	1
2007	outbreak	faeces	2_M	1_roaming	subad	75	0
2007	outbreak	faeces	1_H	2_den-bound	cub	97	0
2007	outbreak	faeces	1_H	2_den-bound	cub	97	1
2007	outbreak	faeces	1_H	2_den-bound	cub	97	1
2007	outbreak	faeces	3_L	1_roaming	ad	75	1
2007	outbreak	faeces	1_H	1_roaming	subad	71	0
2007	outbreak	faeces	2_M	2_den-bound	cub	71	1
2007	outbreak	faeces	2_M	2_den-bound	cub	82	0
2007	outbreak	faeces	1_H	2_den-bound	cub	82	1
2007	outbreak	faeces	1_H	2_den-bound	cub	68	1
2007	outbreak	faeces	1_H	2_den-bound	cub	68	0
2007	outbreak	faeces	1_H	2_den-bound	cub	91	0
2007	outbreak	faeces	1_H	2_den-bound	cub	81	0
2007	outbreak	faeces	2_M	2_den-bound	cub	88	0
2007	outbreak	faeces	2_M	1_roaming	ad	68	1
2007	outbreak	faeces	1_H	1_roaming	subad	68	0
2007	outbreak	faeces	1_H	2_den-bound	cub	80	0
2007	outbreak	faeces	2_M	2_den-bound	cub	79	1
2008	non-outbreak	faeces	2_M	2_den-bound	cub	78	1
2008	non-outbreak	faeces	1_H	2_den-bound	cub	81	0

2008	non-outbreak	faeces	1_H	2_den-bound	cub	81	0
2008	non-outbreak	faeces	1_H	2_den-bound	cub	79	0
2008	non-outbreak	faeces	1_H	2_den-bound	cub	79	0
2008	non-outbreak	faeces	1_H	2_den-bound	cub	77	0
2008	non-outbreak	faeces	2_M	2_den-bound	cub	78	0
2008	non-outbreak	faeces	1_H	2_den-bound	cub	77	0
2008	non-outbreak	faeces	1_H	2_den-bound	cub	79	0
2008	non-outbreak	faeces	1_H	2_den-bound	cub	77	0
2008	non-outbreak	faeces	2_M	2_den-bound	cub	80	0
2008	non-outbreak	faeces	1_H	2_den-bound	cub	77	0
2008	non-outbreak	faeces	2_M	2_den-bound	cub	77	0
2008	non-outbreak	faeces	1_H	2_den-bound	cub	79	1
2008	non-outbreak	faeces	1_H	2_den-bound	cub	76	1
2008	non-outbreak	faeces	2_M	2_den-bound	cub	76	0
2008	non-outbreak	faeces	2_M	2_den-bound	cub	80	0
2008	non-outbreak	faeces	1_H	2_den-bound	cub	71	1
2008	non-outbreak	faeces	1_H	2_den-bound	cub	84	1
2008	non-outbreak	faeces	1_H	1_roaming	subad	82	1
2008	non-outbreak	faeces	3_L	1_roaming	ad	80	0
2008	non-outbreak	faeces	2_M	1_roaming	ad	81	1
2008	non-outbreak	faeces	2_M	1_roaming	subad	81	1
2008	non-outbreak	faeces	1_H	2_den-bound	cub	79	0
2008	non-outbreak	faeces	2_M	1_roaming	subad	83	0
2008	non-outbreak	faeces	1_H	1_roaming	subad	79	0
2008	non-outbreak	faeces	2_M	2_den-bound	cub	79	0
2008	non-outbreak	faeces	1_H	2_den-bound	cub	79	0
2008	non-outbreak	faeces	1_H	2_den-bound	cub	79	0
2008	non-outbreak	faeces	2_M	2_den-bound	cub	79	0
2008	non-outbreak	faeces	2_M	2_den-bound	cub	80	0
2008	non-outbreak	faeces	2_M	2_den-bound	cub	86	0
2008	non-outbreak	faeces	3_L	1_roaming	ad	86	0
2008	non-outbreak	faeces	1_H	2_den-bound	cub	83	0
2008	non-outbreak	faeces	1_H	2_den-bound	cub	77	0
2008	non-outbreak	faeces	2_M	2_den-bound	cub	79	0
2008	non-outbreak	faeces	2_M	1_roaming	ad	84	0
2008	non-outbreak	faeces	2_M	1_roaming	ad	83	0
2008	non-outbreak	faeces	2_M	2_den-bound	cub	83	0
2008	non-outbreak	faeces	1_H	2_den-bound	cub	77	0
2008	non-outbreak	faeces	3_L	1_roaming	ad	86	0
2008	non-outbreak	faeces	2_M	2_den-bound	cub	86	0
2008	non-outbreak	faeces	2_M	2_den-bound	cub	84	0
2008	non-outbreak	faeces	2_M	2_den-bound	cub	84	0
2008	non-outbreak	faeces	2_M	2_den-bound	cub	84	0
2008	non-outbreak	faeces	1_H	2_den-bound	cub	84	0
2008	non-outbreak	faeces	2_M	2_den-bound	cub	83	1
2008	non-outbreak	faeces	2_M	2_den-bound	cub	85	0
2008	non-outbreak	faeces	2_M	1_roaming	ad	85	0
2008	non-outbreak	faeces	1_H	2_den-bound	cub	79	0
2008	non-outbreak	faeces	1_H	2_den-bound	cub	79	0

2008	non-outbreak	faeces	1_H	2_den-bound	cub	85	1
2008	non-outbreak	faeces	3_L	1_roaming	ad	87	0
2008	non-outbreak	faeces	2_M	2_den-bound	cub	79	0
2008	non-outbreak	faeces	3_L	1_roaming	ad	75	1
2008	non-outbreak	faeces	2_M	2_den-bound	cub	79	0
2008	non-outbreak	faeces	2_M	1_roaming	ad	87	1
2008	non-outbreak	faeces	1_H	1_roaming	subad	87	0
2008	non-outbreak	faeces	2_M	2_den-bound	cub	87	0
2008	non-outbreak	faeces	3_L	1_roaming	ad	75	0
2008	non-outbreak	faeces	2_M	2_den-bound	cub	79	0
2009	non-outbreak	faeces	2_M	2_den-bound	cub	81	0
2009	non-outbreak	faeces	1_H	2_den-bound	cub	83	0
2009	non-outbreak	faeces	1_H	2_den-bound	cub	81	0
2009	non-outbreak	faeces	2_M	2_den-bound	cub	76	1
2009	non-outbreak	faeces	1_H	2_den-bound	cub	76	0
2009	non-outbreak	faeces	2_M	1_roaming	ad	82	0
2009	non-outbreak	faeces	2_M	2_den-bound	cub	82	0
2009	non-outbreak	faeces	2_M	1_roaming	ad	79	0
2009	non-outbreak	faeces	1_H	2_den-bound	cub	74	0
2009	non-outbreak	faeces	2_M	2_den-bound	cub	74	0
2009	non-outbreak	faeces	1_H	2_den-bound	cub	74	0
2009	non-outbreak	faeces	1_H	2_den-bound	cub	79	0
2009	non-outbreak	faeces	1_H	2_den-bound	cub	77	0
2009	non-outbreak	faeces	3_L	1_roaming	ad	79	1
2009	non-outbreak	faeces	2_M	2_den-bound	cub	79	0
2009	non-outbreak	faeces	3_L	1_roaming	ad	76	1
2009	non-outbreak	faeces	3_L	1_roaming	ad	83	0
2009	non-outbreak	faeces	2_M	1_roaming	ad	83	1
2009	non-outbreak	faeces	1_H	2_den-bound	cub	82	0
2009	non-outbreak	faeces	1_H	2_den-bound	cub	92	0
2009	non-outbreak	faeces	1_H	2_den-bound	cub	92	1
2009	non-outbreak	faeces	2_M	2_den-bound	cub	92	0
2009	non-outbreak	faeces	2_M	1_roaming	ad	96	0
2009	non-outbreak	faeces	1_H	2_den-bound	cub	96	0
2009	non-outbreak	faeces	1_H	2_den-bound	cub	77	0
2009	non-outbreak	faeces	1_H	1_roaming	subad	87	1
2009	non-outbreak	faeces	2_M	2_den-bound	cub	78	0
2009	non-outbreak	faeces	2_M	2_den-bound	cub	98	0
2009	non-outbreak	faeces	2_M	2_den-bound	cub	99	0
2009	non-outbreak	faeces	1_H	2_den-bound	cub	81	0
2009	non-outbreak	faeces	1_H	2_den-bound	cub	97	0
2009	non-outbreak	faeces	2_M	2_den-bound	cub	97	0
2009	non-outbreak	faeces	1_H	1_roaming	ad	99	0
2009	non-outbreak	faeces	1_H	2_den-bound	cub	99	0
2009	non-outbreak	faeces	2_M	2_den-bound	cub	98	0
2009	non-outbreak	faeces	1_H	1_roaming	ad	80	0
2009	non-outbreak	faeces	1_H	2_den-bound	cub	80	0
2009	non-outbreak	faeces	2_M	2_den-bound	cub	80	0
2009	non-outbreak	faeces	2_M	1_roaming	ad	99	0

2009	non-outbreak	faeces	1_H	2_den-bound	cub	98	0
2009	non-outbreak	faeces	1_H	2_den-bound	cub	98	0
2009	non-outbreak	faeces	2_M	1_roaming	ad	98	1
2009	non-outbreak	faeces	2_M	2_den-bound	cub	78	0
2009	non-outbreak	faeces	2_M	1_roaming	ad	98	0
2009	non-outbreak	faeces	1_H	2_den-bound	cub	102	0
2009	non-outbreak	faeces	1_H	2_den-bound	cub	102	1
2009	non-outbreak	faeces	2_M	1_roaming	ad	102	1
2009	non-outbreak	faeces	2_M	2_den-bound	cub	102	0
2009	non-outbreak	faeces	2_M	1_roaming	ad	102	1
2009	non-outbreak	faeces	1_H	2_den-bound	cub	78	0
2009	non-outbreak	faeces	2_M	2_den-bound	cub	78	0
2010	outbreak	faeces	1_H	2_den-bound	cub	81	1
2010	outbreak	faeces	1_H	1_roaming	ad	110	1
2010	outbreak	faeces	3_L	1_roaming	ad	113	1
2010	outbreak	faeces	3_L	1_roaming	ad	91	0
2010	outbreak	faeces	3_L	1_roaming	ad	116	0
2010	outbreak	faeces	2_M	1_roaming	ad	93	0
2010	outbreak	faeces	2_M	1_roaming	ad	93	0
2010	outbreak	faeces	2_M	1_roaming	ad	96	1
2010	outbreak	faeces	1_H	1_roaming	ad	120	0
2010	outbreak	faeces	3_L	1_roaming	ad	100	1
2010	outbreak	faeces	2_M	1_roaming	ad	118	0
2010	outbreak	faeces	2_M	1_roaming	ad	107	0
2010	outbreak	faeces	1_H	1_roaming	ad	119	0
2010	outbreak	faeces	2_M	2_den-bound	cub	119	0
2010	outbreak	faeces	2_M	1_roaming	ad	120	1
2010	outbreak	faeces	2_M	2_den-bound	cub	115	1
2010	outbreak	faeces	1_H	2_den-bound	cub	115	1
2010	outbreak	faeces	1_H	2_den-bound	cub	121	1
2010	outbreak	faeces	2_M	2_den-bound	cub	115	0
2010	outbreak	faeces	2_M	2_den-bound	cub	115	1
2010	outbreak	faeces	1_H	1_roaming	ad	116	0
2010	outbreak	faeces	1_H	2_den-bound	cub	116	0
2010	outbreak	faeces	1_H	2_den-bound	cub	118	1
2010	outbreak	faeces	1_H	2_den-bound	cub	118	0
2010	outbreak	faeces	2_M	1_roaming	subad	120	1
2010	outbreak	faeces	2_M	2_den-bound	cub	119	1
2010	outbreak	faeces	1_H	2_den-bound	cub	117	0
2010	outbreak	faeces	2_M	1_roaming	ad	116	1
2010	outbreak	faeces	1_H	2_den-bound	cub	116	1
2010	outbreak	faeces	2_M	2_den-bound	cub	117	0
2010	outbreak	faeces	2_M	2_den-bound	cub	117	0
2010	outbreak	faeces	2_M	2_den-bound	cub	117	1
2010	outbreak	faeces	2_M	2_den-bound	cub	117	0
2010	outbreak	faeces	1_H	2_den-bound	cub	116	0
2010	outbreak	faeces	1_H	2_den-bound	cub	116	0
2010	outbreak	faeces	2_M	2_den-bound	cub	117	0
2010	outbreak	faeces	2_M	2_den-bound	cub	117	0

2010	outbreak	faeces	1_H	2_den-bound	cub	116	0
2011	non-outbreak	faeces	2_M	2_den-bound	cub	118	1
2011	non-outbreak	faeces	2_M	2_den-bound	cub	118	0
2011	non-outbreak	faeces	1_H	2_den-bound	cub	118	0
2011	non-outbreak	faeces	1_H	2_den-bound	cub	121	0
2011	non-outbreak	faeces	2_M	2_den-bound	cub	118	0
2011	non-outbreak	faeces	1_H	2_den-bound	cub	121	1
2011	non-outbreak	faeces	2_M	2_den-bound	cub	121	0
2011	non-outbreak	faeces	3_L	1_roaming	ad	121	0
2011	non-outbreak	faeces	1_H	2_den-bound	cub	116	0
2011	non-outbreak	faeces	1_H	2_den-bound	cub	121	0
2011	non-outbreak	faeces	1_H	2_den-bound	cub	121	0
2011	non-outbreak	faeces	1_H	1_roaming	ad	121	0
2011	non-outbreak	faeces	1_H	2_den-bound	cub	121	1
2011	non-outbreak	faeces	1_H	2_den-bound	cub	121	0
2011	non-outbreak	faeces	1_H	2_den-bound	cub	118	0
2011	non-outbreak	faeces	2_M	2_den-bound	cub	121	0
2011	non-outbreak	faeces	2_M	2_den-bound	cub	121	0
2011	non-outbreak	faeces	1_H	1_roaming	subad	124	1
2011	non-outbreak	faeces	2_M	2_den-bound	cub	121	0
2011	non-outbreak	faeces	2_M	2_den-bound	cub	119	0
2011	non-outbreak	faeces	1_H	2_den-bound	cub	119	1
2011	non-outbreak	faeces	2_M	2_den-bound	cub	119	1
2011	non-outbreak	faeces	1_H	2_den-bound	cub	122	0
2011	non-outbreak	faeces	1_H	2_den-bound	cub	119	0
2011	non-outbreak	faeces	1_H	2_den-bound	cub	119	0
2011	non-outbreak	faeces	2_M	2_den-bound	cub	124	0
2011	non-outbreak	faeces	3_L	1_roaming	ad	122	1
2011	non-outbreak	spleen	2_M	2_den-bound	cub	122	0
2011	non-outbreak	faeces	2_M	2_den-bound	cub	121	1
2011	non-outbreak	faeces	2_M	2_den-bound	cub	119	1
2011	non-outbreak	faeces	1_H	2_den-bound	cub	121	0
2011	non-outbreak	faeces	1_H	2_den-bound	cub	121	0
2011	non-outbreak	faeces	2_M	1_roaming	subad	124	0
2011	non-outbreak	faeces	1_H	2_den-bound	cub	121	1
2011	non-outbreak	faeces	3_L	1_roaming	ad	121	0
2011	non-outbreak	faeces	2_M	1_roaming	ad	122	1
2011	non-outbreak	faeces	1_H	1_roaming	ad	123	0
2011	non-outbreak	faeces	1_H	1_roaming	subad	123	0
2011	non-outbreak	faeces	1_H	1_roaming	subad	123	0
2011	non-outbreak	faeces	2_M	2_den-bound	cub	124	0
2011	non-outbreak	faeces	1_H	2_den-bound	cub	123	0
2011	non-outbreak	faeces	1_H	1_roaming	subad	123	0
2011	non-outbreak	faeces	2_M	2_den-bound	cub	123	0
2011	non-outbreak	faeces	1_H	2_den-bound	cub	123	0
2011	non-outbreak	faeces	1_H	2_den-bound	cub	123	0
2011	non-outbreak	faeces	2_M	2_den-bound	cub	124	0
2011	non-outbreak	faeces	1_H	2_den-bound	cub	124	0
2011	non-outbreak	faeces	2_M	2_den-bound	cub	124	0

2011	non-outbreak	faeces	2_M	2_den-bound	cub	124	1
2011	non-outbreak	faeces	1_H	2_den-bound	cub	124	1
2011	non-outbreak	faeces	2_M	2_den-bound	cub	124	1
2011	non-outbreak	faeces	3_L	1_roaming	ad	122	0
2011	non-outbreak	faeces	2_M	1_roaming	ad	124	0
2011	non-outbreak	faeces	1_H	2_den-bound	cub	120	0
2011	non-outbreak	faeces	2_M	2_den-bound	cub	120	0
2011	non-outbreak	lung	1_H	1_roaming	ad	134	0
2011	non-outbreak	faeces	2_M	2_den-bound	cub	123	0
2011	non-outbreak	faeces	2_M	2_den-bound	cub	120	0
2011	non-outbreak	faeces	2_M	2_den-bound	cub	123	0
2011	non-outbreak	faeces	1_H	1_roaming	ad	123	1
2011	non-outbreak	faeces	1_H	1_roaming	ad	120	0
2011	non-outbreak	faeces	1_H	1_roaming	ad	127	0
2011	non-outbreak	faeces	2_M	2_den-bound	cub	123	1
2011	non-outbreak	faeces	2_M	2_den-bound	cub	129	0
2011	non-outbreak	faeces	3_L	1_roaming	ad	120	0
2011	non-outbreak	faeces	2_M	2_den-bound	cub	128	0
2011	non-outbreak	faeces	2_M	2_den-bound	cub	128	0
2011	non-outbreak	faeces	2_M	2_den-bound	cub	129	0
2011	non-outbreak	faeces	1_H	2_den-bound	cub	129	0
2011	non-outbreak	faeces	2_M	2_den-bound	cub	118	0
2011	non-outbreak	faeces	1_H	1_roaming	subad	132	1
2011	non-outbreak	faeces	1_H	2_den-bound	cub	132	1
2011	non-outbreak	faeces	2_M	2_den-bound	cub	118	1
2011	non-outbreak	faeces	3_L	1_roaming	ad	117	0
2011	non-outbreak	faeces	2_M	2_den-bound	cub	126	0
2011	non-outbreak	faeces	2_M	2_den-bound	cub	117	0
2011	non-outbreak	faeces	1_H	2_den-bound	cub	118	0
2011	non-outbreak	faeces	1_H	2_den-bound	cub	125	0
2011	non-outbreak	faeces	2_M	2_den-bound	cub	116	1
2011	non-outbreak	faeces	2_M	2_den-bound	cub	116	1
2011	non-outbreak	faeces	2_M	2_den-bound	cub	134	1
2011	non-outbreak	faeces	2_M	2_den-bound	cub	127	0
2011	non-outbreak	faeces	2_M	2_den-bound	cub	127	0
2011	non-outbreak	faeces	1_H	2_den-bound	cub	134	0
2011	non-outbreak	faeces	2_M	2_den-bound	cub	138	0
2011	non-outbreak	faeces	3_L	1_roaming	ad	123	0
2011	non-outbreak	faeces	3_L	1_roaming	ad	140	0
2011	non-outbreak	faeces	1_H	1_roaming	subad	119	0
2011	non-outbreak	faeces	2_M	1_roaming	ad	119	0
2011	non-outbreak	faeces	2_M	2_den-bound	cub	119	0
2011	non-outbreak	faeces	2_M	2_den-bound	cub	119	0
2011	non-outbreak	faeces	2_M	2_den-bound	cub	126	0
2011	non-outbreak	faeces	2_M	2_den-bound	cub	119	0
2011	non-outbreak	lung	2_M	1_roaming	subad	141	0
2011	non-outbreak	faeces	2_M	2_den-bound	cub	141	0
2011	non-outbreak	faeces	1_H	2_den-bound	cub	119	0
2011	non-outbreak	faeces	1_H	1_roaming	subad	126	0

2011	non-outbreak	faeces	1_H	2_den-bound	cub	142	0
2011	non-outbreak	faeces	2_M	1_roaming	subad	130	0
2011	non-outbreak	faeces	1_H	2_den-bound	cub	140	1
2011	non-outbreak	faeces	1_H	2_den-bound	cub	140	0
2011	non-outbreak	faeces	2_M	2_den-bound	cub	142	0
2011	non-outbreak	faeces	1_H	2_den-bound	cub	142	0
2011	non-outbreak	faeces	2_M	2_den-bound	cub	142	0
2011	non-outbreak	faeces	2_M	2_den-bound	cub	142	0
2011	non-outbreak	faeces	2_M	2_den-bound	cub	142	1
2011	non-outbreak	faeces	2_M	2_den-bound	cub	142	0
2011	non-outbreak	faeces	2_M	2_den-bound	cub	142	0
2011	non-outbreak	faeces	2_M	2_den-bound	cub	142	0
2011	non-outbreak	faeces	1_H	2_den-bound	cub	142	0
2011	non-outbreak	faeces	1_H	2_den-bound	cub	134	0

Year: year in which sample was collected. Outbreak: year categorized as being an outbreak year ("outbreak") or as a non-outbreak year ("non- outbreak"). Samp_Type: type of tissue sample. Contact: social contact rate as defined in the Methods ("1-H" is high social contact rate, "2_M" is medium social contact rate, "3_L" is low social contact rate). Roamer: animal range as defined in the Methods ("1_roaming" for adults and subadults, "2_den-bound" for cubs). Ad_Juv: age categories as defined in the Methods ("cub" 0-12 months old, "subad" 12-24 months old, "ad" older than 24 months). Clan_Size: total number of clan members on the day of sampling. Sapovirus_Pcr: Dependent variable reporting the outcome of genetic diagnostic (1 is presence of Sapovirus, 0 is absence).
