# Supplementary material for: Divergent Sapovirus Strains and Infection Prevalence in Wild Carnivores in the Serengeti Ecosystem: A Long-Term Study
Source: PLoS One. 2016 Sep 23;11(9):e0163548. doi: 10.1371/journal.pone.0163548 (PMC5035092; doi:10.1371/journal.pone.0163548)
Supplement: S2 Table — (PDF) [file pone.0163548.s002.pdf]

Supporting Information

Divergent sapovirus strains and infection prevalence in wild carnivores in the Serengeti ecosystem: a long-term study

Ximena A Olarte-Castillo, Heribert Hofer, Katja V Goller, Vito Martella, Patricia D Moehlman, Marion L East

S2 Table: Data set for the analysis of repeatedly sampled spotted hyenas

Individual	Sample Sequence	Interval Days	RT-PCR
1	first	826	neg-pos
1	second		
2	first	814	neg-pos
2	second		
3	first	932	neg-neg
3	second		
4	first	848	neg-pos
4	second		
5	first	1156	pos-pos
5	second		
6	first	775	pos-neg
6	second		
7	first	1768	neg-pos
7	second		
D	first	26	neg-neg
D	second	1698	neg-pos
D	third		
8	first	1370	pos-pos
8	second		
9	first	76	pos-pos
9	second		
10	first	385	neg-neg
10	second		
11	first	893	neg-neg
11	second		
12	first	993	neg-pos
12	second		
13	first	683	pos-pos
13	second		
A	first	124	neg-neg
A	second	10	neg-neg
A	third		
14	first	848	pos-neg
14	second		
15	first	551	neg-pos
15	second		

16	first	432	pos-neg
16	second		
K	first	755	pos-pos
K	second	5	pos-neg
K	third		
J	first	118	neg-pos
J	second	715	pos-pos
J	third		
17	first	626	pos-neg
17	second		
18	first	158	neg-pos
18	second		
E	first	9	neg-neg
E	second	969	neg-pos
E	third		
19	first	149	neg-pos
19	second		
C	first	23	neg-neg
C	second	131	neg-neg
C	third	563	neg-neg
C	fourth		
20	first	3	neg-neg
20	second		
21	first	86	neg-neg
21	second		
22	first	19	neg-pos
22	second		
H	first	16	neg-neg
H	second	20	neg-neg
H	third	352	neg-pos
H	fourth		
23	first	108	pos-neg
23	second		
24	first	101	neg-neg
24	second		
25	first	64	neg-neg
25	second		
26	first	29	neg-pos
26	second		
27	first	8	pos-neg
27	second		
B	first	19	neg-neg
B	second	82	neg-neg
B	third	188	neg-neg
B	fourth		
I	first	6	neg-neg
I	second	11	neg-neg

I	third	22	neg-pos
I	fourth		
28	first	166	neg-neg
28	second		
29	first	57	neg-neg
29	second		
30	first	39	neg-neg
30	second		
L	first	3	pos-neg
L	second	99	neg-neg
L	third		
31	first	90	neg-neg
31	second		
32	first	2740	pos-neg
32	second		
33	first	911	neg-pos
33	second		
34	first	300	neg-pos
34	second		
35	first	1705	neg-neg
35	second		
36	first	54	neg-neg
36	second		
37	first	25	neg-pos
37	second		
38	first	1214	neg-pos
38	second		
39	first	89	neg-pos
39	second		
40	first	22	neg-pos
40	second		
41	first	1173	neg-pos
41	second		
42	first	596	neg-neg
42	second		
43	first	183	neg-pos
43	second		
44	first	275	neg-neg
44	second		
45	first	371	pos-pos
45	second		
46	first	45	neg-neg
46	second		
47	first	180	pos-neg
47	second		
48	first	9	neg-pos
48	second		

49	first	81	neg-neg
49	second		
50	first	143	neg-neg
50	second		
M	first	16	pos-neg
M	second	29	neg-neg
M	third		
51	first	68	neg-neg
51	second		
52	first	35	pos-pos
52	second		
53	first	95	neg-neg
53	second		
54	first	21	neg-neg
54	second		
F	first	35	neg-neg
F	second	110	neg-pos
F	third		
55	first	54	neg-pos
55	second		
56	first	84	neg-neg
56	second		
57	first	1810	neg-neg
57	second		
58	first	1227	neg-neg
58	second		
59	first	703	neg-neg
59	second		
60	first	1093	neg-pos
60	second		
61	first	1522	pos-pos
61	second		
62	first	2105	pos-neg
62	second		
63	first	192	neg-pos
63	second		
64	first	286	neg-neg
64	second		
65	first	1712	pos-pos
65	second		
66	first	1026	pos-neg
66	second		
N	first	143	pos-neg
N	second	38	neg-neg
N	third		
67	first	124	neg-neg
67	second		

68	first	106	neg-pos
68	second		
69	first	120	neg-pos
69	second		
70	first	5	neg-neg
70	second		
71	first	755	neg-neg
71	second		
72	first	41	neg-neg
72	second		
73	first	64	pos-pos
73	second		
74	first	129	neg-neg
74	second		
75	first	189	neg-neg
75	second		
G	first	20	neg-neg
G	second	5	neg-pos
G	third		
O	first	27	pos-neg
O	second	35	neg-neg
O	third	41	neg-neg
O	fourth		
76	first	10	neg-pos
76	second		

RT-PCR: Sapovirus RNA infection status: positive (Pos), negative (Neg). Individuals A-O the same animals listed in Table 2. Individuals numbered 1-76 were only sampled on two occasions and are not included in Table 2.
